# Range extension and re-assessment of *Eugenia stirpiflora* (O.Berg) Krug & Urb., an endemic Myrtaceae to the Virgin Islands

**DOI:** 10.3897/BDJ.13.e175549

**Published:** 2025-12-16

**Authors:** Sara Bárrios, Clare Weaver, Eleanor Gibney, Thomas Mark Heller, Martin A Hamilton, Marcella Corcoran, Keith Grant, Juan Viruel, Amy Barker, Nancy Woodfield-Pascoe, Colin P. Clubbe

**Affiliations:** 1 Royal Botanic Gardens Kew, London, United Kingdom Royal Botanic Gardens Kew London United Kingdom; 2 Virgin Islands Rare Plant Initiative (VIRPI), Saint John, Virgin Islands (USA) Virgin Islands Rare Plant Initiative (VIRPI) Saint John Virgin Islands (USA); 3 Independent Researcher, Saint John, Virgin Islands (USA) Independent Researcher Saint John Virgin Islands (USA); 4 South Carolina Botanical Garden at Clemson University, Clemson, United States of America South Carolina Botanical Garden at Clemson University Clemson United States of America; 5 National Parks Trust Virgin Islands, Road Town, Virgin Islands (British) National Parks Trust Virgin Islands Road Town Virgin Islands (British)

**Keywords:** Caribbean flora, conservation status, distribution, endemism, Myrtaceae, new records, Red List, Virgin Islands

## Abstract

**Background:**

The Caribbean archipelago is a global biodiversity hotspot, with high levels of endemism and a threatened flora. The British Virgin Islands and the US Virgin Islands, two archipelagos situated in the Caribbean Sea are no exception. Despite many decades of floristic investigation, continuing botanical surveys across the region are uncovering previously unrecorded plant populations and improving our understanding of the geographic ranges of locally restricted threatened flora. This study aims to re-evaluate the latest conservation status of *Eugenia stirpiflora* by examining its current spatial distribution, population numbers, main threats and conservation actions needed to ensure the species' long term survival.

**New information:**

Thought to be endemic to the island of St. John in the US Virgin Islands (USVI), *E. stirpiflora* (Myrtaceae), was discovered on the island of Virgin Gorda in the British Virgin Islands (BVI) in 2020, extending its geographic range to a new territory. Despite this wider distribution, the species' extent of occurrence (20 km^2^) and area of occupancy (12 km^2^) are still highly restricted. On St. John (USVI), this species is recorded from Europa Bay, including the White Cliffs area, Minna Hill and Upper John's Folly. On Virgin Gorda, the species is only known from Fanny Hill, north of Gorda Peak. The total number of mature individuals ranges between 1,535 and 1,665, with the species' largest population occurring on Europa Bay (St. John) with a number of mature individuals between 1,500 and 1,630. On the island of Virgin Gorda, the species' habitat is declining due to expansion of urban development and uncontrolled grazing by feral ungulates. On St. John, the suitable habitat and the number of individuals are declining due to grazing by free-roaming livestock and feral ungulates, illegal trail clearing and insect pests. Based on these threats, two locations were recognised. Simultaneously, prolonged periods of drought, caused by climate change, are affecting both locations uniformly. This species, previously evaluated on the IUCN Red List as Critically Endangered (CR) under its synonym, *Eugenia earhartii*, is here re-evaluated as Endangered (EN), based on Criteria B1ab(iii,v)+2ab(iii,v); C2a(ii) according to the IUCN Red List Categories and Criteria.

## Introduction

**Figure 1.**
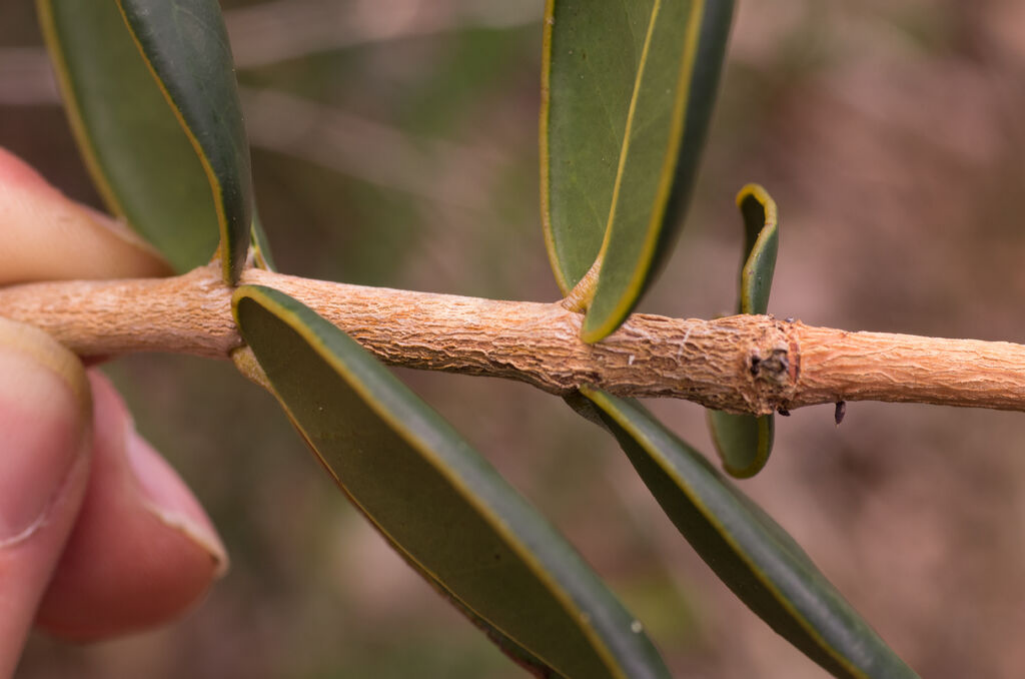
Leaves of *Eugenia stirpiflora* on a tree from Fanny Hill, Virgin Gorda. The leaves are coriaceous with strongly revolute margins (Photo: Thomas Mark Heller, RBG Kew).

**Figure 2.**
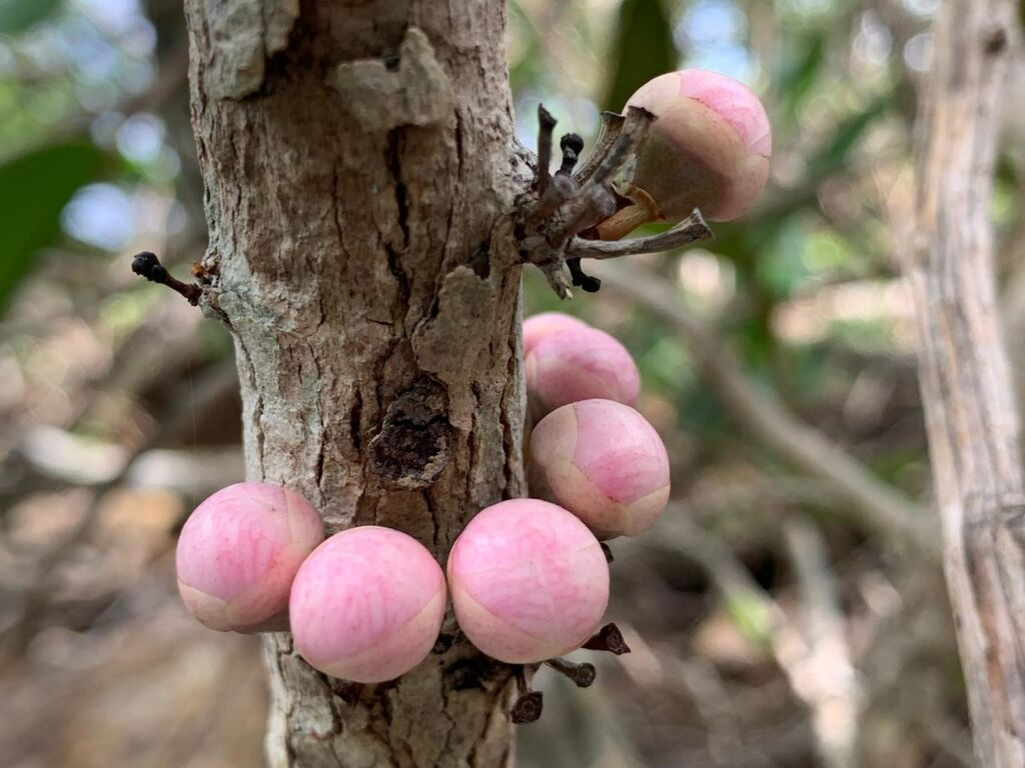
Flower buds of *Eugenia stirpiflora*, emerging from a tree on St. John (USVI) (Photo: Clare Weaver, VIRPI).

**Figure 3.**
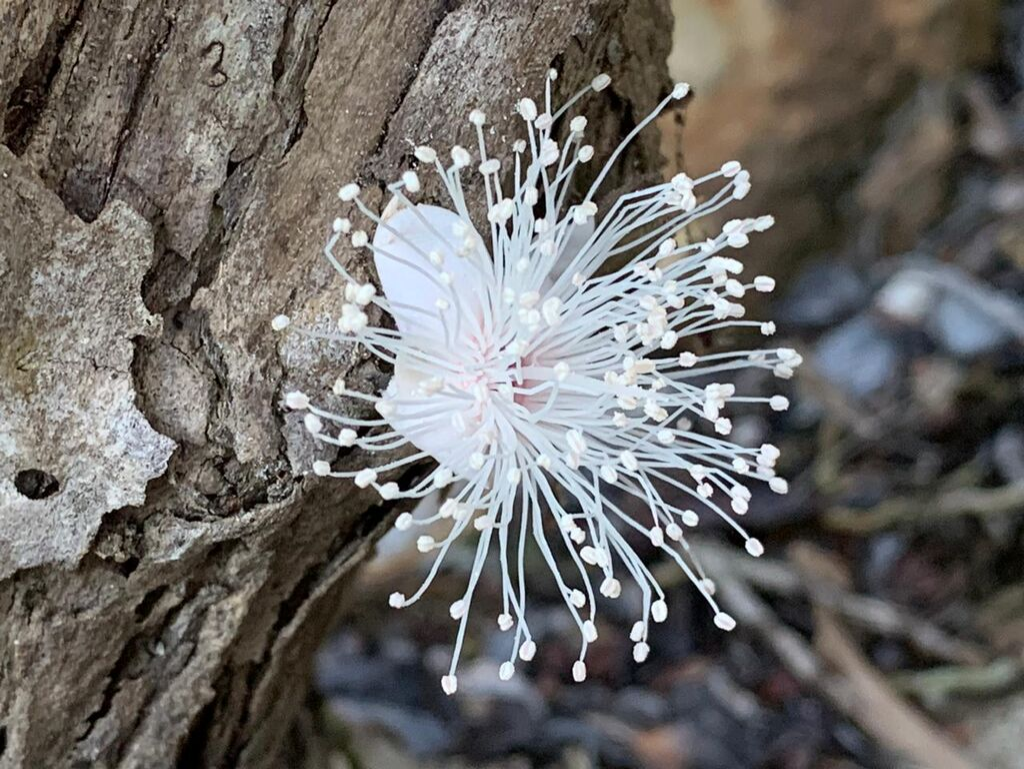
A cauliflorous flower of *Eugenia stirpiflora* on Fanny Hill, Virgin Gorda (BVI) (Photo: Sara Bárrios, RBG Kew).

**Figure 4.**
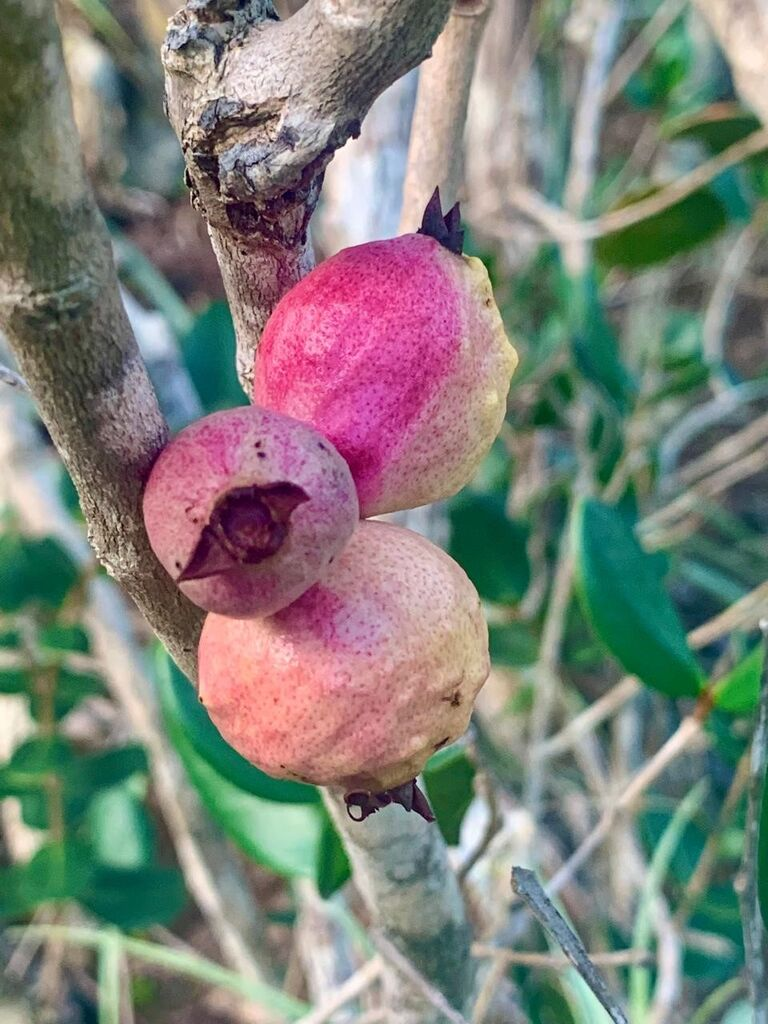
Fruits of *Eugenia stirpiflora* on a St. John tree (Photo Clare Weaver, VIRPI).

**Figure 5.**
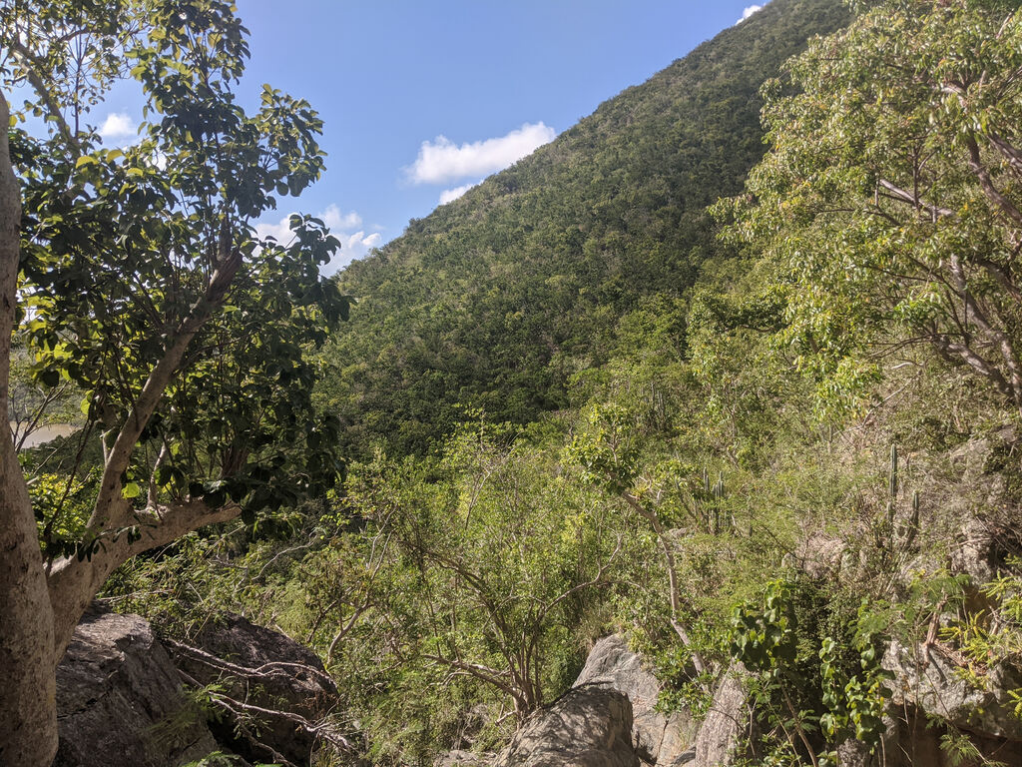
Undisturbed tropical dry forest is the preferred habitat for *Eugenia stirpiflora* (Photo: Thomas Mark Heller, RBG Kew).

*Eugenia stirpiflora* (O.Berg) Krug & Urb. grows as a shrub or small tree up to eight metres tall. The bark is grey and thick, becoming gnarled and shedding in irregular patches. The leaves are sessile and coriaceous. Blades are ovate to elliptical, with a rounded apex. The leaf margin is strongly revolute (Fig. 1) similar to the leaves of the close relative *E. sessiliflora* Vahl. The flowers are cauliflorous, emerging directly from the bark on short pedicels (Fig. 2). Flowers are white with a pink tinge (Fig. 3). The fruit is globose to oblate (Fig. 4) (Acevedo-Rodriguez 1996, Flickinger et al. 2022). This species grows in relatively undisturbed tropical dry forest, in association with other native plant species (Fig. 5) between 20 to 214 metres high (Acevedo-Rodriguez 1996, Flickinger et al. 2022).

Thought to be restricted to the island of St. John in the USVI (Acevedo-Rodriguez 1996, Lindsay et al. 2015), this species was discovered on the island of Virgin Gorda in the BVI in February 2020 during a survey of Fanny Hill (Dani Sanchez et al. 2020). Vouchers (T.M. Heller, #1038, K000817252, K!), DNA samples (J. Viruel, #JV22-001, K!) and cuttings for ex-situ collections (G. Gregg, #11, BVI!), collected between 2020 and 2022, have now confirmed the identity of this species at this location (Heller 2022, Barrios 2023). Although administered by different countries, the British Virgin Islands and the US Virgin Islands lie in close geographical proximity to each other and, floristically, are part of the Puerto Rican Bank flora (Acevedo-Rodriguez 1996).

## *In situ* and *ex situ* conservation initiatives

**Figure 6.**
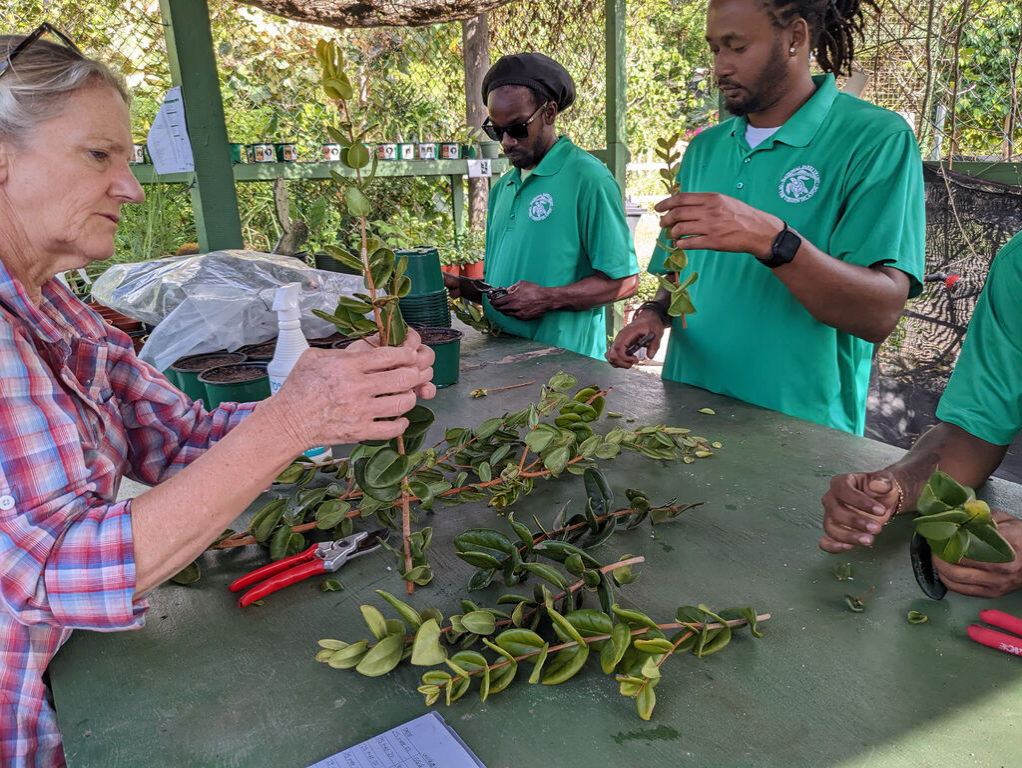
*Eugenia stirpiflora* being propagated by cuttings at J.R. O'Neal Botanic Garden on Tortola, BVI (Photo: Thomas M. Heller, RBG Kew).

Trials to asexually propagate this species by cuttings were undertaken at the J.R. O'Neal Botanic Garden on Tortola, BVI in 2022. Cuttings were collected from the recently discovered wild plants at Fanny Hill, on Virgin Gorda, BVI. The cutting trials used semi-hardwood and hardwood materials and two different media types which were available in the nursery. All cuttings were dusted with rooting hormone powder (Fig. 6). Pots were placed in a misting unit to maintain a high relative humidity, until the material developed roots. No cuttings survived long-term from these initial propagation trials, suggesting that this species might not be easily propagated by these methods. Further propagation research is needed.

**Figure 7.**
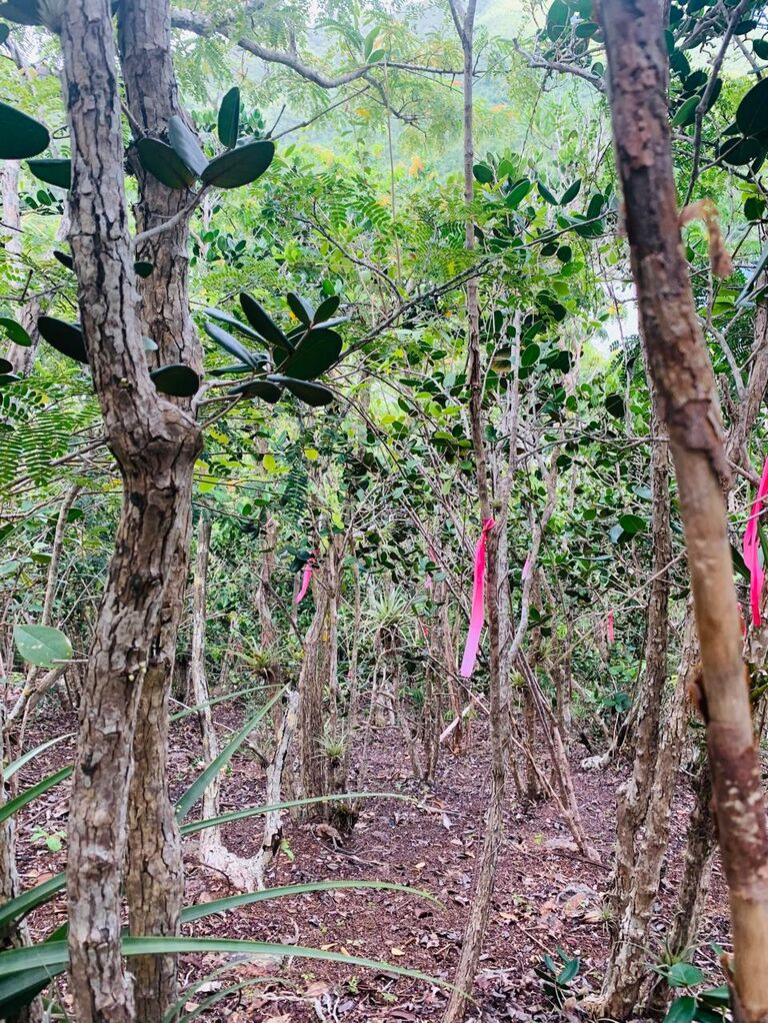
Monitoring plot with tagged *Eugenia stirpiflora* trees on St. John, USVI (Photo: Clare Weaver, VIRPI).

In the USVI, the Virgin Islands Rare Plant Initiative (VIRPI) has undertaken regular population monitoring activities since 2012 at Europa Bay, including the White Cliffs area. Monitoring includes collecting measurements and photographic data and also observing fruiting cycles (Fig. 7). The VIRPI is working to collect seeds from each individual (maternal lines), to be germinated and conserved at a living gene bank on St. Croix, USVI and shared with other regional institutions.

### Conservation genetics of Eugenia stirpiflora

We assessed the genetic diversity of the BVI population of *E. stirpiflora*. A total of ten individuals were sequenced and genotyped, using SNPs derived from target capture of 353 nuclear loci. The observed heterozygosity (*H*_O_) was 0.227 ± 0.003, while the expected heterozygosity (*H*_E_) was 0.278 ± 0.003, indicating moderate levels of genetic diversity within the population. The inbreeding coefficient (*F*_IS_) was 0.080 ± 0.009, suggesting a slight deficit of heterozygotes relative to Hardy–Weinberg expectations.

The moderate levels of genetic diversity observed in *Eugenia stirpiflora* (*H*_E_ = 0.278) are comparable to those reported in other narrowly endemic plant species studied with target capture SNPs (Liang et al. 2025, Razanamaro et al. 2025). Similarly, Slimp et al. (2021) reported *H*_E_ values between 0.23 and 0.31 in several range-restricted taxa using a comparable Angiosperms353-based pipeline. The inbreeding coefficient for *E. stirpiflora* (*F_IS_* = 0.080) suggests only mild inbreeding pressure, aligning with values typical for outcrossing species with small census sizes. These preliminary findings suggest that the BVI population of *E. stirpiflora* retains appreciable genetic variation despite its geographically restricted distribution.

The analysis of a single population limits inference on overall species-level diversity and genetic structure; therefore, we recommend the inclusion of additional samples from USVI to cover the species’ distribution range. Such broader sampling will enable an assessment of genetic connectivity, population differentiation and potential local adaptation, all of which are critical for providing information for conservation strategies under ongoing habitat disturbance and climate change.

## Methods

Information on the species' distribution was gathered from digitised herbarium collections held at K, field observations between 2020 and November 2026 and from previous assessments by Ray (2018), Rivers and Ray (2021). Field surveys have also provided additional information on habitat, main threats and population sizes. The extent of occurrence and the area of occupancy were calculated with GeoCAT (Bachman et al. 2011). The conservation assessment evaluation followed the IUCN Red List Categories and Criteria and guidelines (IUCN Standards and Petitions Committee 2024).

For the conservation genetic studies, DNA was extracted from silica gel-dried leaf tissue using a modified Cetyltrimethylammonium bromide (CTAB) protocol following Doyle and Doyle (1987), with all extractions conducted at the Jodrell Laboratory, Royal Botanic Gardens, Kew, UK. Genomic library preparation used 200 ng of input DNA and followed the protocol outlined in Viruel et al. (2019), incorporating half reaction volumes from the NEBNext® Ultra™ II DNA Library Prep Kit for Illumina® (New England Biolabs, Ipswich, MA, USA). Indexing was performed using NEBNext® Multiplex Oligos for Illumina® (Dual Index Primer Sets I and II) and purification was achieved using AMPure XP magnetic beads. Enrichment for target loci employed the Angiosperms353 bait set (Johnson et al. 2019, Baker et al. 2022), following the myBaits® manual version 5.03 (Arbor Biosciences). The hybridisation pool consisted of all ten libraries combined in equimolar concentrations. DNA concentrations were assessed with a Quantus™ fluorometer (Promega Corp.) and fragment size distribution was verified using the Agilent 4200 TapeStation (Agilent Technologies, Santa Clara, CA, USA). Libraries were sequenced on an Illumina HiSeq platform at Macrogen (Seoul, South Korea) using 150 bp paired-end reads. Raw genomic data were deposited in the ENA repository prior to publication.

Raw reads were assessed for quality using FastQC (Andrews, 2010) and MultiQC (Ewels et al. 2016). Adapter sequences and low-quality bases (LEADING:30, TRAILING:30) were removed using Trimmomatic (Bolger et al. 2014). Cleaned reads were aligned to the Mega353 target reference (McLay et al. 2021) using BWA (Li and Durbin 2009) and assembled de novo with SPAdes (Bankevich et al. 2012) via the HybPiper pipeline (Johnson et al. 2016). Introns were recovered using the intronerate option in HybPiper.

To enable SNP discovery, the longest intron per gene was selected to create a reference file. Variant calling followed the pipeline developed by Slimp et al. (2021), modified according to Razanamaro et al. (2025) to integrate Base Quality Score Recalibration (BQSR) using GATK (McKenna et al. 2010). Reads were aligned to the intron reference using BWA, PCR duplicates were removed with GATK and variants were called with GATK’s variant discovery tools (Poplin et al. 2018). SNPs were filtered using the following quality parameters: QD < 5.0, FS > 60.0, MQ < 40.0, MQRankSum < -12.5 and ReadPosRankSum < -8.0. To reduce linkage, SNPs were pruned in PLINK (Chang et al. 2015) using the --indep 50 5 2 setting.

Genetic diversity indices, including allelic richness, observed (*H*_O_) and expected heterozygosity (*H*_E_) and inbreeding coefficient (*F*_IS_), were calculated in GeneAlEx (Peakall and Smouse 2012).

Sequence data generated has been deposited in the European Nucleotide Archive (ENA), with the accession number PRJEB96302.

## Species Conservation Profiles

### Eugenia stirpiflora

#### Species information

Scientific name: Eugenia stirpiflora

Species authority: (O.Berg) Krug & Urb.

Synonyms:

*Eugenia earhartii* Acev.-Rodr. in Brittonia 45: 133 (1993)

*Myrciaria stirpiflora* O.Berg in Linnaea 30: 702 (1861)

Common names: Earhart's Stopper

Kingdom: Plantae

Phylum: Tracheophyta

Class: Magnoliopsida

Order: Myrtales

Family: Myrtaceae

Taxonomic notes: According to Flickinger et al. (2022), the review of their type specimens and similar descriptions of *Eugenia stirpiflora* and *Eugenia earhartii*, which were both described from St. John, suggests they are the same species and supports their synonymisation.

Region for assessment: Global

#### Editor & Reviewers

##### Reviewers

Reviewers: Beech, E.; Wen, L.Y.

##### Editor

Editor: Barrios, S.; Ray, G.; Rivers, M.

#### Geographic range

Biogeographic realm: Neotropical

Countries: Virgin Islands, BritishVirgin Islands, U.S.

Map of records (Google Earth): Suppl. material 1

Basis of EOO and AOO: Observed

Basis (narrative): The extent of occurrence (EOO) was calculated to be 20 km^2^ and the area of occupancy to be 12 km^2^, based on a 2 × 2 km cell size, using GeoCAT (Bachman et al. 2011).

Min Elevation/Depth (m): 30

Max Elevation/Depth (m): 257

Range description: *Eugenia stirpiflora* is a shrub or small tree, endemic to the British (BVI) and the US Virgin Islands (USVI). Thought to be restricted to the island of St. John in the USVI (Acevedo-Rodriguez 1996, Lindsay et al. 2015), this species was discovered on the island of Virgin Gorda in the BVI in 2020 (Dani Sanchez et al. 2020). Vouchers and subsequent surveys conducted in 2022, 2023 and 2024 have now confirmed the identity of this species at this location (Heller 2022, Barrios 2023). On St. John in the USVI, the species is known to be extant at three localities: Europa Bay, including the White Cliffs area, Minna Hill and Upper John's Folly. On Virgin Gorda in the BVI, this species is known only from Fanny Hill, north of Gorda Peak (Suppl. material 2).

#### Extent of occurrence

EOO (km2): 20

Trend: Unknown

Causes ceased?: Unknown

Causes understood?: Unknown

Causes reversible?: Unknown

Extreme fluctuations?: No

#### Area of occupancy

Trend: Unknown

Causes ceased?: Unknown

Causes understood?: Unknown

Causes reversible?: Unknown

Extreme fluctuations?: No

AOO (km2): 12

#### Locations

Number of locations: 2

Justification for number of locations: The number of locations was calculated to be two, considering the expansion of urban development, including road construction activities on Virgin Gorda (BVI) and feral livestock on St John (USVI), which are the main threats to this species and are impacting all individuals equally.

Trend: Unknown

Extreme fluctuations?: No

#### Population

Number of individuals: 1,535-1,665

Trend: Decline (observed)

Justification for trend: On Virgin Gorda (BVI), no reduction in the numbers of individuals has been observed; however, individuals found at the Fanny Hill are likely to be impacted by activities associated with the construction and maintenance (e.g. road widening) of Nail Bay road over recent decades. On St. John (USVI), illegal trail clearing within the Virgin Islands National Park resulted in the death of five to ten mature individuals. A stem borer has also been observed to affect the health of and, in some cases, cause mortality of individuals at Europa Bay on St. John (Flickinger et al. 2022). As a result of these ongoing threats, a decline in the overall population is observed.

Basis for decline: (a) direct observation(c) a decline in area of occupancy, extent of occurrence and/or quality of habitat

Causes ceased?: No

Causes understood?: Yes

Causes reversible?: Unknown

Extreme fluctuations?: No

Population Information (Narrative): The estimated total number of mature individuals ranges between 1,535-1,665 mature individuals, with the species' largest population occurring on Europa Bay, including the White Cliffs area (St. John, USVI), with 1,500 to 1,630 individuals. The smallest population occurs on Upper John's Folly on St. John (USVI) with only one known mature individual and one sapling. Despite extensive fieldwork, only one subpopulation is known on Virgin Gorda, comprising fewer than 10 individuals including mature trees and saplings. Habitat destruction and illegal trail clearing have led to the loss of mature individuals across this species range.

#### Subpopulations

Number of subpopulations: 2

Trend: Unknown

Justification for trend: 

Extreme fluctuations?: No

Severe fragmentation?: No

#### Habitat

System: Terrestrial

Habitat specialist: No

Habitat (narrative): This species grows in relatively undisturbed tropical dry forest, in association with other native plant species, including *Parasenegalia muricata* (L.) Seigler & Ebinger, *Psychilis macconnelliae* Sauleda, *Byrsonima lucida* (Mill.) DC., *Tillandsia utriculata* L., *Oplonia spinosa* (Jacq.) Raf., *Scolosanthus versicolor* Vahl, *Piptocoma antillana* Urb. and *Ipomoea eggersiana* Peter. On Fanny Hill (Virgin Gorda, BVI), small saplings have been observed growing by the side of the road, but the forest above the road cut is relatively intact.

Trend in extent, area or quality?: Decline (observed)

**Figure 8.**
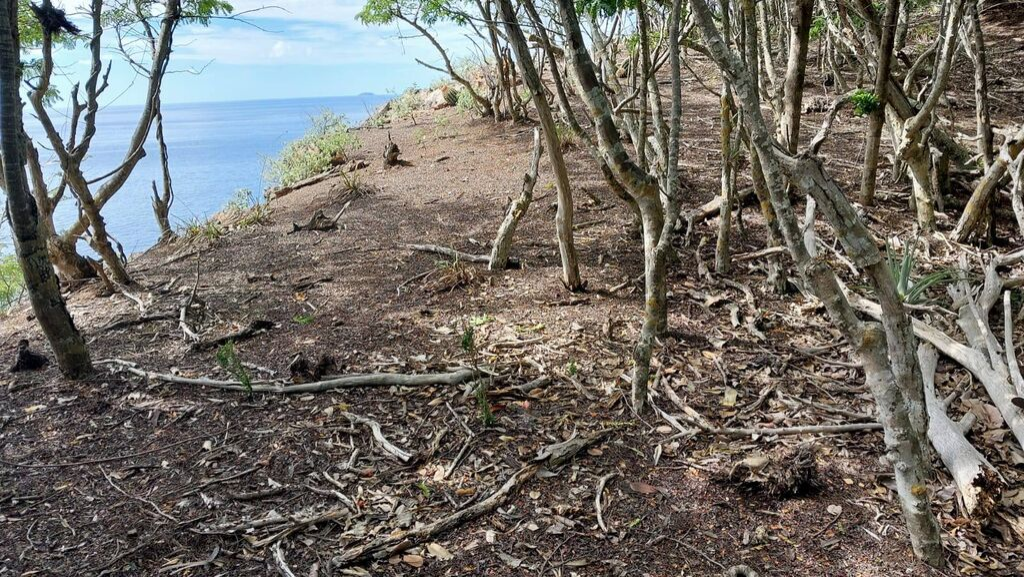
Habitat degradation caused by feral goats and deer at White Cliffs area on St John (Photo: Sara Bárrios, RBG Kew).

Justification for trend: On Virgin Gorda (BVI), the suitable habitat of this species is declining due to expansion of urban development, including road opening and maintenance activities. On St. John (USVI), the suitable habitat is also declining due to the opening of illegal trails within the Virgin Islands National Park. Additionally, suitable habitat is declining due to the presence of free-roaming livestock and feral ungulates across this species' range, which heavily modify the vegetation, topsoil conditions (Fig. 8) and recruitment of the flora, as well as directly feeding on this species.

Figure(s) or Photo(s): Fig. 8

##### Habitat

Habitat importance: Major Importance

Habitats: 1.5. Forest - Subtropical/Tropical Dry

#### Ecology

Generation length (yr): 0

Dependency of single sp?: Unknown

Ecology and traits (narrative): The generation length of this species is unknown. Flowers have been observed to emerge between March and October. This species is very slow-growing. Plants observed on St. John that are 10 years old have only reached 2 metres in height, with stems only about 2 cm in diameter, except at the very base. Seedlings and saplings have been observed growing in all subpopulations.

#### Threats

**Figure 9.**
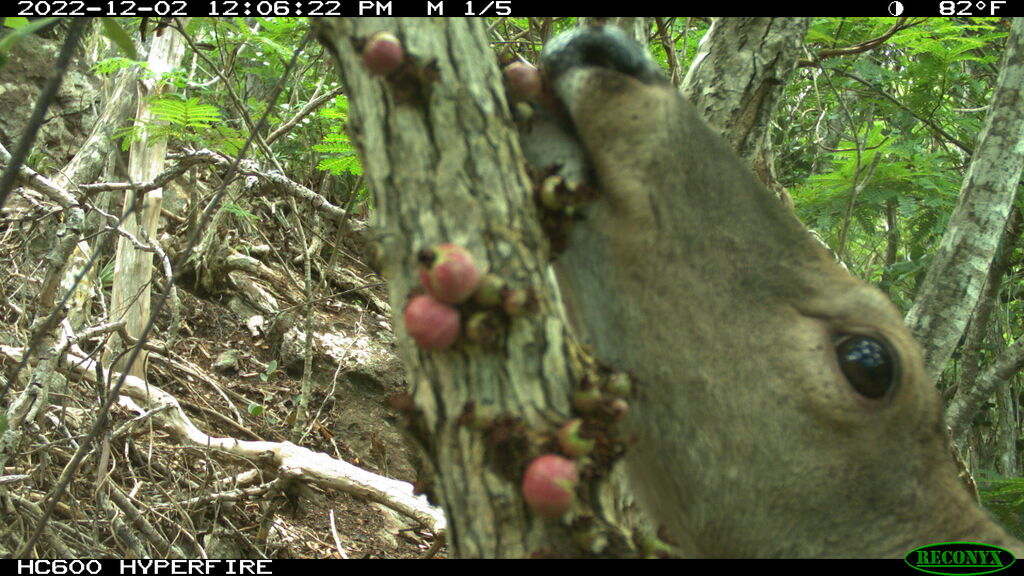
White-tailed deer (*Odocoileus virginianus*), a non-native and invasive species on St John, predating the fruits of *Eugenia stirpiflora*. (Photo: U.S. National Parks Service, camera-trap image).

Justification for threats: There are several threats which are impacting this species across its range, in a variety of ways. This species' suitable habitat is being negatively impacted by the expansion of urban development, including road opening and maintenance and recreation activities, such as the opening of illegal hiking trails. These unauthorised pathways have also contributed to a reduction in the number of mature individuals. Free-roaming feral livestock and ungulates are also impacting this species' habitat and predating its fruits (Fig. 9), affecting the species population numbers. A stem borer has been observed on St John (USVI), impacting the number of mature individuals at this locality. Prolonged periods of drought, caused by climate change, are affecting both locations equally.

##### Threats

Threat type: Ongoing

Threats: 4.1. Transportation & service corridors - Roads & railroads6.1. Human intrusions & disturbance - Recreational activities8.1. Invasive and other problematic species, genes & diseases - Invasive non-native/alien species/diseases8.6. Invasive and other problematic species, genes & diseases - Diseases of unknown cause11.2. Climate change & severe weather - Droughts

#### Conservation

Justification for conservation actions: This species occurs within protected areas only on the island of St. John (USVI), where it is found within the boundaries of the Virgin Islands National Park. Fanny Hill, the only known locality for this species in the BVI, is not protected by any formal legal designation, though it falls within the Central Virgin Gorda Tropical Important Plant Area (TIPA) (Dani Sanchez et al. 2021) and it is under considerable development pressure. Protection for the locality and habitat where this species occurs in the BVI is urgent. Although unsuccessful in establishing mature plants, trials to asexually propagate this species by cuttings have taken place at the J.R. O'Neal Botanic Garden on Tortola (BVI) in 2022. In the USVI, the VIRPI has set up regular monitor activities at Europa Bay area since 2012. The VIRPI is working to collect seeds from each individual, to be germinated and conserved at a living gene bank on St. Croix (USVI) and other regional institutions. Areas of suitable habitat across the British and US Virgin Islands remain under-surveyed and should be targeted in future botanical survey work. Conservation genetic studies should be expanded to include samples from both territories. A cross-territory management plan should be established to ensure coordinated efforts in protecting the species across different geographical and political regions.

##### Conservation actions

Conservation action type: In Place

Conservation actions: 1.1. Land/water protection - Site/area protection3.4.1. Species management - Ex-situ conservation - Captive breeding/artificial propagation

##### Conservation actions

Conservation action type: Needed

Conservation actions: 1.2. Land/water protection - Resource & habitat protection2.2. Land/water management - Invasive/problematic species control3.4. Species management - Ex-situ conservation5.1. Law & policy - Legislation4.3. Education & awareness - Awareness & communications

#### Other

Justification for use and trade: There are no known uses for this species.

Justification for ecosystem services : Although this species' ecosystem services are unknown, the dry forest habitat, where this species is found, is essential for climate regulation and habitat maintenance.

##### Use and trade

Use type: National

##### Ecosystem services

Ecosystem service type: Very important

Ecosystem services: 4. Climate Regulation8. Habitat Maintenance

##### Research needed

Research needed: 1.2. Research - Population size, distribution & trends1.3. Research - Life history & ecology2.2. Conservation Planning - Area-based Management Plan3.4. Monitoring - Habitat trends

Justification for research needed: Research is needed to investigate the population genetics of both sub-populations and to fully understand the ecology of this species.

## Conclusion

Botanical surveys for rare and threatened plants remain an important activity to fill gaps in our knowledge of the distribution and conservation status of this important group of plants (Clubbe et al. 2020). Ongoing survey efforts continue to yield new plant discoveries on these islands, enhancing our understanding of their botanical diversity (Trejo-Torres et al. 2014, Barrios et al. 2021) More broadly, botanical surveys are also leading to the discovery of new species (Acevedo-Rodriguez 1993, Sukhorukov et al. 2021) and to re-discovery of previously thought extinct species (Baker et al. 2014, Abeli et al. 2021) across many parts of the world.

Species with highly restricted ranges, like *E. stirpiflora*, spanning multiple countries require coordinated regional conservation strategies to prevent extinction, as collaborative management efforts across national boundaries — which species' distributions do not recognise — are essential for effective protection throughout their entire range. These strategies must include public engagement activities to raise awareness of these unique species and their conservation needs. Further genetic study should include samples from across the species range. We propose that a regional species action plan for *E. stirpiflora* represents the optimal approach for maintaining viable populations and preserving suitable habitat, enabling this threatened species to thrive.

Conclusion

## Supplementary Material

EF131AF1-0B6F-521E-90CF-19F6BF82A8C9 10.3897/BDJ.13.e175549.suppl1 Supplementary material 1 Eugenia stirpiflora occurrences Data type occurrences Brief description *Eugenia stirpiflora* occurrences. Data from Herbarium collections and human observations. File: oo_721688.kml https://binary.pensoft.net/file/721688 Barrios, S.

73C05F49-606A-5D15-8121-FC81513EF60C 10.3897/BDJ.13.e175549.suppl2 Supplementary material 2 Eugenia stirpiflora occurrences - IUCN Point Data Standard Data type occurrences Brief description *Eugenia stirpiflora* occurrences. Data from Herbarium collections and human observations. File formatted according to the IUCN Data Point Standards. File: oo_1442454.csv https://binary.pensoft.net/file/1442454 Barrios, S.
